# Letter from Dr. Harlan—No. IV

**Published:** 1839-07-13

**Authors:** 


### FOREIGN CORRESPONDENCE.

### LETTER FROM DR. HARLAN—NO. IV.

*M. Velpeau's Operations—Hôpital St. Louis—Messrs. Jobert, Biett, and Lugol—Hôpital du Midi—M. Ricord—Hôpital Neckar—M. Civiale—M. Dubois' Cæsarean Operation—Prevalence of Suicide—M. Magendie's Course of Experimental Physiology—Dupuytren's Anatomical Museum—M. Azous' Lectures on Anatomy—Visit to the Catacombs—British Medical Society of Paris—The late Emeute—Removal of a Scrofulous Tumour, by M. Velpeau—M. Magendie's Recent Observations on the Functions of the Spinal Nerves.*

Paris, May 20th, 1839.

To the Editors of the Medical Examiner:

Gentlemen—I should not have taken leave, in my last, of M. Velpeau, without referring, as further testimony of his industry and research, to his numerous volumes on surgery, anatomy and obstetrics. Although yet a middle-aged man, he has published fifteen or sixteen reputable works. He is probably better acqainted with the surgical improvements of the Americans than any of his Parisian confrêres. He occasionally quotes the names of Warren, Mott, Physick, and Dr. Rhea Barton. I recently witnessed him (or as they call it in Paris, "*assisted*" him) in six minor operations during one of his daily lectures, from nine to ten, A. M. One was a lad with fistula lachrymalis, whose ears he severely boxed for not holding his head still. The lachrymal tube, so much used by Dupuytren, has gone out of fashion. M. Senchelles says he has removed ten or fifteen of these tubes from the lachrymal ways of patients treated some years since by Dupuytren, and which had become, in a manner, encysted. M. V. introduces a bougie of ivory, softened by means of an acid solution, which is removed when the fistula is sufficiently dilated. Another case which presented itself the same morning was that of an old man, with fistula in ano, in which a laughable incident occurred, showing M. V.'s predilection for cutting. After the ordinary section, the surgeon amused himself in trimming the cut edges of the wound, very deliberately, when, all at once, a large, projecting mole on the back of the patient caught his eye. It was too tempting a bait. He immediately transferred his scissors to it, and incised it at its base, very much to the discomforture of the patient and merriment of the class, one of whom remarked that M. V. *cultivated* a patient's *acquaintance* very much like the gardener cultivated his plants, by lopping off all offensive parts and useless exuberances.

The Hôpital St. Louis presents a valuable school of instruction, and its reputation would attract a greater number of students, but that its location, so far from their ordinary haunts, renders attendance there inconvenient. This hospital was originally destined for diseases of the skin and for all contagious maladies, but at present it is by no means confined to these departments. M. Jobert is an expert operator, and a gentleman of very pleasing address. His opportunities, as also those of MM. Biett and Lugol, of studying diseases of the skin, are certainly unsurpassed by those of any surgeons in the world, and yet I cannot but express my disappointment in their success, when examining the results of their practice in the more inveterate classes of these maladies. Many cases of "Dartre," or tetter, they pronounce incurable, and their treatment of these is merely palliative. Their treatment of ordinary cases is very prolix. They neglect, in an astonishing manner, the state of the patient's constitution, their chief attention being mainly directed to the local affection. Their remedies consist of diet, baths, mercury, sulphur, hydrosulphuret of ammonia, iodine and its compounds. M. Lugol prescribes his tincture of iodine in every case of disease of the skin that comes into his ward. There is no hospital in Paris so generally attended as the "Hôpital du Midi," or "Des Veneriens," nor is there any lecturer more popular than Dr. *Ricord*, who, I think, is a native of Baltimore, and who, for a long time, served in the shop of Dr. Rousseau, of Philadelphia. He now enjoys a high reputation, and the best practice of of any surgeon of Paris. He has also published one of the most important works on the venereal disease that has appeared of late years, in which his descriptions are very lucid, and his deductions for the most part unimpeachable. His hospital is at a great distance from the part of the town which I inhabit, and I have only occasionally enjoyed the opportunity of listening to his interesting instructions; but during my visit to Paris, five years ago, I made his hospital the pivot of my movements for some time. His practice then was much more extensive in his hospital than at present, all the female patients having been removed to another hospital, where the surgeon himself is not privileged to introduce students. Dr. Ricord lectures as well in the English as in the French language. He is also a gentleman of most amiable and accessible manners, but it is very much to be regretted that he occasionally forgets the dignity due to his station and condescends to resort to vulgar witticisms, for the temporary applause of the more frivolous portion of his class; but it must be confessed that the numerous anecdotes be has collected in his immense private practice are at once the most ridiculous specimens of national peculiarities, and display the inconceivable manner in which mankind, under the influence of a vitiated imagination, may become demoralized, debased and "denaturé." It is true, also, that to these very national peculiarities referred to, are due some of the most important improvements in the treatment of venereal. The enthusiasm of Dr. Ricord occasionally surpasses all bounds. Illustrating his lecture the other morning with a beautiful imitation of a diseased bone, he declared that "God himself might be jealous of the artist who made this preparation!" Where any doubt exists as to the primary, secondary, or tertiary nature of the disease, Dr. R. is in the habit of inoculating the patient from his own sore, as the surest test, as the *primitive* chancre is alone capable of being reproduced. Dr. R. has satisfied himself by repeated experiments that the disease is in no manner aggravated by repeated inoculation. Inasmuch as Dr. R. has detailed most of his improvements in his work above alluded to, and is about to publish his more recent experience, it would be inexpedient in me to occupy more of your time on this subject.

I have met with some disappointment in my visits to Hôpital Neckar. As far as I have observed, not a single case for lithotritie has presented itself in this hospital during the past winter—not that Dr. Civiale's well earned reputation has, in any degree, diminished; his private practice is extensive and his reputation in the treatment of the diseases of the urinary organs is unsurpassed. Very few cases of stone in the bladder present themselves for treatment in the hospitals, during the winter. Dr. Mott informs me that he witnessed two cases of the high operation for the stone, by Le Roy, during the last month. Both patients, he thought, would die, as did two others of a similar nature, which he witnessed last year. Dr. Civiale is better known and esteemed by foreign physicians, who visit Paris, than any other of his confrères, a distinction which he claims by superior merit, frankness, liberality, and attentive politeness to strangers. His ménage is on a most liberal scale; and besides his soirées, every Tuesday, his repeated dinner parties are most munificently furnished, as I know from my own experience.

Monsieur Dubois, son of the late Accoucheur to the late Empress, some weeks since produced considerable sensation in Paris, by the performance, for the sixth time, of the Cæsarean operation, the result being fatal to the mother in every case. The subject of the present operation was an ugly, deformed Cretan, who has been hawked about the country as a show, by a tall, giraffe-looking man, who has, by this means, obtained a beggar's subsistence, and who, by his own confession, fearing that death or accident might deprive him of his capital stock in trade, determined, if possible, to obtain a second edition, which would resemble the mother as closely as possible; but to his great disappointment, and which poorly repaid his solicitous attentions to the mother, whilst confined to the hospital, the infant proved an epitome of the father.

The operation had been anticipated for several weeks, and had been discussed even in the drawing rooms of Paris. It finally took place, without an hour's notice; the room was crowded to excess, and a crowd was excluded at the door. I was not among the fortunate ones who gained admission. Mr. Van Buren was more successful. He thought that the intestines of the unfortunate patient, already worn out by previous suffering, were too long exposed. She died in two or three days from peritonitis. The infant still lives.

One of my chief objects being to witness as great a variety of surgical operations as possible, I began to look out for a case of *decapitation.* I soon learned by the newspapers that two candidates awaited this climax of surgery. We made the necessary arrangements to "assist" at the operation, but were effectually disappointed, *suicide* having become fashionable about this time, among the "chevaliers d'industrie." One of the criminals, *Soufflade*, swallowed three drachms of powdered arsenic in going from court to prison, and died in horrible agony, early the next morning. The other, *Le Sage*, waited until the day previous to that of his execution, and hung himself in his cell—thus, by a strange contradiction, desperadoes who were not ashamed to murder a defenceless woman, for a few francs, were too modest to mount the guillotine. A female has since been condemned to death for the murder of her lover. When on examination, her intention of committing suicide was known by the detection of a pair of scissors, hid beneath her armpit. A robber was taken the other morning in our immediate neighborhood, in the act of carrying off some furniture. He shot a bullet through his head in descending the stairs, which, with the frequent cases in the Morgue taken from the Seine, proves that suicide is a contagious affection.

This *Soufflade* above mentioned was thoroughly autopsied, and Orfila detected poison in almost every tissue of the body, not excepting the bones; but I am informed that he subsequently detected arsenic in other bones. What curious results are sometimes produced when ultimate principles are set free and recombined in a red hot crucible!

M. Magendie's spring course of experimental physiology commenced in the beginning of April. I seldom fail of "assisting" at his murders. At his first lecture, a basket full of live rabbits, a glass receiver full of frogs, two pigeons, an owl, several tortoises and a pup were the victims ready to lay down their lives for the good of science! His discourse was to explain the functions of the fifth pair of nerves. The facility was very striking with which the Professor could cut the nerve at its origin, by introducing a sharp instrument through the cranium, immediately behind and below the eye. M. Magendie drew the attention of the class to several rabbits, in which the fifth pair of nerves had been divided several days before. They were all blind of one eye, a deposition of lymph having taken place in the cornea, from inflammation of the eye always following the operation alluded to, although the eye is by this section deprived of all sensibility. Monsieur M. has not only lost all feeling for the victims he tortures, but he really likes his business. When the animal squeaks a little, the operator grins; when loud screams are uttered, he sometimes laughs outright. The professor has a most mild, gentle and amiable expression of countenance, and is in the habit of smoothing, fondling, and patting his victim, whilst occupied with preliminary remarks; and the rabbit either looks him in the face, "or licks the hand just raised to shed his blood." During another lecture, in demonstrating the functions of the motive and sensative fibres of the spinal nerves, he laid bare the spinal cord in a young pup, and cut one bundle after another, of nerves. The phrenological developments of the professor belie those of physiognomy. His skull is enormously broad between and behind the ears. Living dissection is as effectual a mode of teaching as it is revolting, and in many cases the experiments are unnecessarily cruel, and too frequently reiterated; but so long as the thing is going on, I shall not fail to profit by it, although I never wish to see such experiments repeated.

Since my last visit to the French metropolis, a most magnificent temple has been erected and consecrated to anatomical science. I allude to the Museum of Dupuytren, near the School of Medicine. With all his sins, and he had many grievous ones to answer for, Monsieur D. was among the greatest of surgeons. If his labours had been confined to the collection, preservation, and useful display of this invaluable collection, he would have ranked among the greatest benefactors of his profession. By practice, aided by speculation, under the auspices of his friend, Rothschild, Baron Dupuytren amassed great wealth. He bequeathed enough to erect a superb museum and richly endow it. The collection not only includes all the pathology resulting from his own perseverance and indefatigable research, but is much enlarged by the liberal contributions of his êléves and friends. Among the striking objects is a preparation of Baron Larrey's, the skull of a soldier of the Egyptian army, in which remains the iron ramrod, which entered the cranium, above the nose, and passed out at the occiput, resulting in the death of the patient after some days, and which resisted every effort at abstraction.

There is one entire cabinet of skeletons, exemplifying the effects of mollities ossium, or deficiency of osseous matter, producing every possible form of distortion in subjects of different ages; the adjoining cabinet is filled with skeletons, displaying the effects of an entirely opposite morbid state, wherein many specimens are completely ossified, the skeleton forming but a single piece. Representations, in wax, of *Glanders* or *la Morve*, in various stages as it occurs in the human species, no case of which, it is asserted, has been cured, occupy another cabinet.

Every form of malignant tumour and ulcer, together with all cuticular diseases, are represented with shocking fidelity; in fine, every morbid function of the body has here its faithful analogue. Since the completion of this lasting monument to the memory of Dupuytren, the celebrity of the famed museum of the School of Medicine has departed.

Among the improvements in anatomy, though not a very recent one, I ought to mention the gratification that I experienced in attending the lectures of Dr. Azous, who, for the benefit of the fastidious, has succeeded in divesting anatomy of all its disgust and horror, and rendered it a pursuit even for ladies, a number of the most respectable of whom were constant attendants of his demonstrations, which are made entirely from artificial subjects, in which each portion is separate, marked with names or numbers, and in which there is one advantage it possesses over the real subject, that all the relative positions of muscle, tendon, nerve, bloodvessel, and bone, are beautifully displayed;—it is not pretended, of course, that, to the practical surgeon, an artificial pasteboard composition, however accurately it may resemble nature, can ever be substituted for the familiarity of dissection with the flesh and blood of a dissecting room, especially in Paris, where whole subjects can be purchased for two dollars, in great profusion—I might say, in too great a profusion—dissectors become careless, the rooms are damp, cold, and filthy, and many a laborious student has contracted disease here which has sent him to his long home.

As a subject not altogether irrelevant to a professional letter, I must notice here, a visit I lately made to the catacombs under a part of Paris. These renowned receptacles of the dead have been for some time entirely closed to the public, and permission to enter in particular cases is obtained with much delay and difficulty. After long anticipating a permit from the efforts of several influential friends, and finding myself no nearer the accomplishment of my object, I addressed a letter in my own name to M. Rambuteau, Pair de France, et Prêfet du department de la Seine, which, in a few days, brought me a favourable reply. I soon organized a party of seven, chiefly of my compatriots, and at 7 o'clock, P. M., on the 2d of May, we found ourselves at the iron gates opening into the catacombs, a short distance beyond the barriere St. Jacques; the descent is by a stone staircase of fifty feet, winding around a central pillar. We had passed but a short distance under the walls of the city, when we arrived at the gate which opens into the receptacles of the bones. We shall not attempt to detail what has already been so faithfully and frequently depicted; but our attention was soon arrested by the great number of skulls, probably as many as fifteen or twenty per cent., in which the os frontis was divided by the sagittal suture, extending down to the nose. Our guide inducted us into another inclosure, denominated the Salle de Minèralogie, where specimens of the rocks and minerals obtained in the excavations of the catacombs are neatly and scientifically arranged; but we were more interested in a collection of diseased bones, which had been here preserved from among the thousands that had been here collected, and some of which we took the liberty of abstracting. Continuing our route through sloping paths, we descended altogether about one hundred and fifty feet beneath the surface of the city, where there is a fine spring of water on a level with the river Seine. We next visited a curious model of Port Mahon, in the Isle of Minorca, carved out of the calcareous rock, by a French labourer employed in the excavation of the catacombs, and who had been a prisoner many years in Port Mahon Castle.

An ardent phrenologist might spend many days in admiring the curious forms of skulls in this immense collection, numerous specimens of which have been transferred by Gall, Cuvier, Dupuytren, &c., to their own cabinets. It is asserted that the remains of at least three millions of human beings are here congregated; we concluded that out of so many skulls a few would not be missed, and acted on this idea. This interesting visit occupied us about one hour and a half, when we returned by the road we entered.

Early in May, the British Parisian Medical Society opened its summer session; it is constituted chiefly of young practitioners from London, Scotland, and Ireland, who are studying or pursuing their profession, some temporarily, some permanently, in Paris. After an introductory discourse by Sir Robert Chermside, the president, some interesting observations were made on "fissure of the rectum," a most troublesome affection, by no means uncommon in the Parisian hospitals, and which, in the opinion of many of the best surgeons here, is curable only by a most painful operation, viz., section of the sphincter ani, as in the operation of fistula in ano; this treatment is always resorted to by Velpeau.

One of the debaters remarked that he had seen strongly marked cases of this affection successfully treated in the German hospitals, by means of a strong solution of sulphate of zinc. The same disease occurs in Dublin, but very rarely in London, many of whose eminent practitioners confessed that they had never met with it. I have never seen a similar affection during my long service in the Philadelphia hospital, and have observed recorded but two cases in America; these, I think, occurred in Massachusetts. A paper was next read by Dr. Acton, the Secretary, on "Primary Venereal Chancre of the Mouth." Dr. Ricord being present, his remarks were elicited, when he delivered a discourse in English, of an hour's length, in his usual facetious and felicitous manner. He stated, among other facts, as a proof of the high value to be placed on the use of the speculum uteri, an instance that had come under his observation that very day.

A gentleman called at his office with an exceedingly painful sore on the tongue. Mr. R. assured him that it was a venereal chancre. The patient declared that to be impossible, as the Dr. might satisfy himself by his own observations, inasmuch as the only female with whom he cohabited was then in the adjoining chamber. It is true, continued Dr. R., that by the ordinary superficial examination, no appearance of disease could be detected in this patient; but by having recourse to the speculum, a chancre was revealed occupying the cervix uteri!

Surgery began about this time to become a little stale in Paris, when a most unexpected event filled several of the hospitals with the wounded, of a most interesting nature. On the afternoon of May 12th, a mob of armed men suddenly appeared in the neighbourhood of the Hôtel de Ville and Palais de Justice; they killed the soldiers at several stations, and seized their arms. When they appeared before the Prêfecture de Police, they were repulsed by bullets. They gained possession of the open space before the Hôtel de Ville, and endeavoured to fortify it with barricades. They were soon dislodged by the municipal guard, with some slaughter on both sides,—but the mob, always getting the worse of it, they were driven from barricade to barricade, but not without desperate resistance. I found myself at the hospitals by times next morning. M. Roux, at the Hôtel Dieu, was in high spirits; all the wards displayed numerous cases of gunshot wounds, of every variety of kind and extent. The dead-house contained thirty bodies of the factious, who had died during the night, of their wounds; they, of course, formed interesting subjects for dissection. Next day, M. Breschet was about amputating an arm of one of the rebels at the shoulder-joint, when a juge de paix appeared, and insisted on his right to examine the patient judicially. This was loudly opposed by the students, the judge desisted, the operation was postponed by the surgeon, and that day the doors of the hospital were hermetically sealed against all but the surgeons and their assistants. At the Hospital St. Louis there were also numerous cases of the wounded, ten or twelve deaths the next morning, and two operations during the day for amputation at the shoulder-joint. In La Charité, only three cases of similar wounds occurred. Some of the wounded were peaceable citizens, led on by curiosity to expose themselves to the shot,—some were shopkeepers, endeavouring to save their goods,—but most of them appeared to be men of the lowest order, perfectly reckless and careless of life, who exposed themselves boldly to the fire of overwhelming majorities, and died without fear. I stopped into the *Morgue* on my return from the Hôtel Dieu; there were nine bodies exposed, some of them terribly mangled, and all probably shot dead; they included persons of different ages, from eighteen to fifty—some with countenances of tigers. The day after, these bodies were removed, and replaced by nine more, several of whom had evidently died of hæmorrhage.

Of the military, it is admitted that at least forty were killed, including some officers of high rank, and a few of the National Guard.

As yet, nothing has been elicited from the numerous prisoners taken with arms in their hands, or from the wounded in the hospitals, respecting the object of the rioters.

On the 13th of May the mob again rallied, and attacked the Polytechnic School; they were kept at bay by the students within, and soon dispersed by the military, with the loss of several lives.

Up to the present date, (18th May,) more than seventy of the insurgents are dead of their wounds; more than one hundred are prisoners, and the wounded under treatment exceeds one hundred.

I believe it is a rule in medicine and surgery, acted upon by the most experienced of our American practitioners, "When we know not precisely what to do, to do nothing at all." I was reminded of this axiom this morning, when M. Velpeau, at the hospital La Charité, removed a tumour, nine or ten inches in circumference, from the axilla of an interesting girl of twelve or thirteen years of age, for a carcinomatous disease, when, on examination, it proved, to his own satisfaction, to be a scrofulous tumour; thus subjecting a patient to a most painful operation, without the chance of a permanent cure, from a mere love of *cutting*.

Magendie communicated to the Institute to-day some new observations on the functions of the spinal nerves. The nerves of sensation and the motor nerves, he says, are compound in their functions, and are associated with each other at their extremities. When a section of the nerves of sense is made near the spinal column, and irritation is applied at the lower portion, sensation and motion are produced; the same phenomena occur when the motor nerves are similarly treated, whilst not the least sensation is occasioned by irritating that portion of the bisected nerves which terminate at the nervous centre, or the brain.

I intended to allude, in the present letter, to the "*Jardin des Plantes*," as a source of *medical* instruction; also, to speak of some of the hospitals for the insane, and of the several orthopêdique institutions in Paris and its vicinity,—but an opportunity of despatch occurring, I am obliged to close.

